# Cloning and expression of a novel α-1,3-arabinofuranosidase from *Penicillium oxalicum* sp. 68

**DOI:** 10.1186/s13568-018-0577-4

**Published:** 2018-04-02

**Authors:** Yanbo Hu, Xuecui Yan, Han Zhang, Jiaqi Liu, Feng Luo, Yingying Cui, Weiyang Wang, Yifa Zhou

**Affiliations:** Jilin Province Key Laboratory on Chemistry and Biology of Natural Drugs in Changbai Mountain, School of Life Sciences, Northeast Normal University, Changchun, 130024 China

**Keywords:** α-l-Arabinofuranosidase, Glycoside hydrolase family 62, *Penicillium oxalicum* sp. 68, Sugar beet arabinan, Arabinoxylan

## Abstract

The discovery and creation of biocatalysts for plant biomass conversion are essential for industrial demand and scientific research of the plant cell wall. α-1,2 and α-1,3-l-arabinofuranosidases are debranching enzymes that catalyzing hydrolytic release of α-l-arabinofuranosyl residues in plant cell wall. Gene database analyses shows that GH62 family only contains specific α-l-arabinofuranosidases that play an important role in the degradation and structure of the plant cell wall. At present, there are only 22 enzymes in this group has been characterized. In this study, we cloned a novel α-1,3-arabinofuranosidase gene (*poabf62a*) belonging to glycoside hydrolase family 62 from *Penicillium oxalicum* sp. 68 and expressed it in *Pichia pastoris*. The molecular mass of recombinant PoAbf62A was estimated to be 32.9 kDa. Using *p*-nitrophenyl-α-l-arabinofuranoside (*p*NPαAb*f*) as substrate, purified PoAbf62A exhibited an optimal pH of 4.5 and temperature of 35 °C. Results of methylation and ^13^C NMR analyses showed that PoAbf62A was exclusively α-1,3-arabinofuranosidase, specific for cleavage of α-1,3-arabinofuranosyl residues, and with the absence of activity towards α-1,2-arabinofuranose and α-1,5-arabinofuranose. Therefore, PoAbf62A exhibits high activity on sugar beet arabinan and wheat arabinoxylan, because their branched side chain are decorated with α-1,3-arabinofuranose. On the other hand, there is a lack of activity with linear-α-l-1,5-arabinan and xylan that only contained α-l-1,5-arabinofuranose or β-1,4-xylose. The α-1,3-arabinofuranosidase activity identified here provides a new biocatalytic tool to degrade hemicellulose and analyze the structure of plant cell walls.

## Introduction

Glycoside hydrolases are a broad group of enzymes that hydrolyse the glycosidic bond of saccharides and their derivatives (Scigelova et al. 1999). Presently, about 148 glycoside hydrolase families have been reported, based on sequence and structural similarities in the CAZy database (Lombard et al. 2014). There are widely used in the bio-processing industry and for the structural analysis of polysaccharides from the plant cell wall because of high efficiency, specificity, and low pollution (Himmel et al. 2007). The α-l-arabinofuranosidases (ABFs) are enzymes that hydrolyze terminal non-reducing end α-l-arabinofuranoside residues in α-l-arabinosides. They are classified into families GH2, GH3, GH10, GH43, GH51, GH54 and GH62, with the ABFs of families GH43 and GH62 belonging to GH-F that have a five-bladed β propeller arrangement and an inverting mechanism of hydrolysis (Vandermarliere et al. 2009; Wang et al. 2014; Maehara et al. 2014). The GH62 family only contains α-l-arabinofuranosidases of fungal and bacterial origin. Based on ABFs’ substrate specificity, they can be divided into type A and B (Pitson et al. 1996). ABFs of type A only act on arabinoxylooligosaccharides and *p*-nitrophyl-α-l-arabinofuranoside, whereas ABFs of type B have equivalent activities on oligosaccharide and polysaccharides (Van Laere et al. 1997). All GH62 family belong to type B, because they are more active on polymeric arabinoxylan that is singly or doubly substituted with α-l-1,2-Ara*f* and α-l-1,3-Ara*f* residues (Kaur et al. 2015; Hashimoto et al. 2011). They have little activity on low molecular-weight substrates, such as unbranch arabinan and *p*NPαAra*f* (Wilkens et al. 2017; Sakamoto et al. 2011). ABFs have become known from their various industrial applications, such as biotransformation of plant residues, food processing, and bleaching of pulp (Saha 2000; Numan and Bhosle 2006). Aside from this, ABFs can completely degrade hemicelluloses and pectins in combination with other accessory hemicellulases and pectic enzymes (Margolles-Clark et al. 1996; Spagna et al. 1998). The ABFs from GH62 family are promising candidates for use in the structural analysis of polysaccharides and in regulating the chemical xylan to saccharify xylan for bio-refinery applications.

Other than d-xylose, l-arabinose is the second most abundant pentose in polysaccharides from the plant cell wall that is widely populated with arabinan, arabinoxylan, arabinogalactan and rhamnogalacturonan I (Seiboth and Metz 2011; Caffall and Mohnen 2009). The backbones of these polysaccharides often contain arabinofuranose side chains. Arabinan is composed of an α-1,5-l-arabinofuranose backbone with α-1,3-l-arabinofuranose and α-1,2-l-arabinofuranose side chains (Sakamoto and Kawasaki 2003). Arabinoxylan consists of β-1,4-d-xylan in which arabinose residues are substituted of O-2 or O-3 sites of the xylan backbone (Kormelink et al. 1993). Arabinogalactan is present on the backbone of β-1,3-galactose-linked with α-l-arabinofuranose and β-l-arabinopyranose side chains (Ponder and Richards 1997). In addition, the rhamnogalacturonan-I backbone is also substituted with various oligosaccharides, such as arabinooligosaccharides and galactan oligosaccharides (Gruppen et al. 1993). l-Arabinose is an important sugar in the food and biofuel industries, as well as in pharmaceutics (Amore et al. 2012), and it has excellent potential as prebiotics and inhibition of sucrose absorption (Altamimi et al. 2006; Seri et al. 1996). The production of l-arabinose by α-l-arabinofuranosidases is efficient and environmentally friendly. As the main component of hemicellulose, xylan also has potential applications in food industry. Because the side chains of xylan are often decorated with α-l-arabinofuranose (Song et al. 2012), it is necessary to hydrolyze arabinose residues efficiently using α-l-arabinofuranosidases to produce xylan (Gao et al. 2011). Thus, it is essential to obtain a variety of α-l-arabinofuranosidases to prepare oligosaccharide fragments and analyze the structures of polysaccharides. In this work, we have explored the biochemical characterization of a novel α-1,3-arabinofuranosidase (PoAbf62A) from *P. oxalicum* sp. 68, that can specially remove α-l-1,3-Ara*f* from sugar beet arabinan. This work provides a new tool for industrial preparation of polysaccharides, as well as for the structural analysis of polysaccharides.

## Materials and methods

### Strains and reagents

*Penicillium oxalicum* sp. 68 with the collection number CGMCC 7.328 in China General Microbiological Culture Collection Center was isolated from Chang bai mountain soil (Jilin Province, China). DH5α, *E. coli* BL 21 (DE3), *P. pastoris* GS115, pET-32a (+) and pPIC9K (Novagen, Madison, WI, USA) were used as host and expression vectors, respectively. *p*NP-α-l-arabinofuranoside (*p*NPαAra*f*), *p*NP-α-l-arabinopyranoside (*p*NPαAra*p*) *p*NP-β-galactopyranoside (*p*NPβGal), *p*NP-α-galactopyranoside (*p*NPαGal), *p*NP-β-xylopyranoside (*p*NPβXyl), *p*-nitrophenyl-β-glucopyranoside (*p*NPβGlc), *p*NP-α-glucopyranoside (*p*NPαGlc) were purchased from Sigma (St. Louis, MO, USA). Sugar beet l-arabinan, linear-1,5-α-l-arabinan, and wheat arabinoxylan (low viscosity) were obtained from Megazyme International Ireland Ltd. (Wicklow, Ireland). All of other chemicals and reagents were analytical grade.

### Construction of plasmids and strains

The total RNA was extracted from *P. oxalicum* sp. 68, the mycelia were frozen in liquid nitrogen, homogenized with mortar and pestle, and extracted with Trizol. The cDNA encoding for α-1,3-arabinofuranosidase was reverse by reverse-transcriptase-PCR using a reverse-transcriptase (Cat, No, M531A, Promega) and oligo-dT primer. According to the amino acid sequence which was reported in a previous paper (Liu et al. 2013; Li et al. 2013), primers having restriction sites for *Nde*I (GGAATTCCATATGGCTGGTACCCTTGCGAG) and *Eco*RI (CGGAATTCTTACTAGTGCTTCAGGGTGAGA) were used for gene amplification. PCR was performed using DreamTaq Green PCR Master Mix (Thermo Scientific), and the program was following: 95 °C for 30 s, 30 cycles of 95 °C for 10 s, 66 °C for 45 s, 72 °C for 1 min 30 s, and finally extension at 72 °C for 10 min. The PCR product and pET-32a (+) were digested with *Nde*I and *Eco*RI, and gene was ligated with pET-32a (+) to generate the recombinant plasmid pET32a-*poabf62a*. All enzymes used were from New England Biolab (Beverly, MA). The restriction enzyme digestions, ligations and transformations were performed as the suppliers’ recommendations. For PoAbf62A expression, *E. coli* BL21 (DE3) cells harboring pET32a-*poabf62a* were grown in 100 ml of LB broth with 100 μg/ml ampicillin at 37 °C. When the OD_600 nm_ reached 0.5, the culture was induced with 0.5 mM IPTG and then grown for 16 h at 25 °C. For eukaryotic expression system, the specific primers were *poabf62a*-F (GACCTACGTAGCTGGTACCCTTGCGAGTGC) and *poabf62a*-R (CGGAATTCTTACTAGTGCTTCAGGGTGAGA), under the following conditions: denaturing at 94 °C for 30 s, annealing at 62 °C for 45 s, and amplification at 72 °C for 1 min 30 s, 30 cycles. The product of PCR was ligated with pPIC9K (*Sna*BI/*Eco*RI). Approximately 5 μg of the recombinant plasmid (*poabf62a*-pPIC9K) was linearized by *Sal*I and electrotransformed into GS115 cells. Electroporation and selection of transformants were carried out by using MD and G418. The selected clone was cultured in BGMY medium at 30 °C for 3 days, with methanol being supplemented (0.5%) every 24 h during the induction period. Cells were harvested by centrifugation at 8000 rpm for 10 min, and the crude enzyme was found in the supernatant.

### Purification of recombinant PoAbf62A

PoAbf62A was purified from the culture supernatant of recombinant *P. pastoris* GS115, and enzyme activity was assayed throughout the purification steps. The culture medium (200 ml) was centrifuged at 8000 rpm for 10 min, and the supernatant collected and precipitated with 80% ammonium sulfate, dissolved and dialyzed against 20 mM Na-acetate buffer (pH 4.5). The protein was passed through a 10 × 1.6 cm Phenyl Sepharose 6 Fast Flow (high sub) column (GE Healthcare) which contained ammonium sulfate at a saturated concentration of 30%. Adsorbed proteins were then eluted by gradient elution with ammonium sulfate (from 30, 20, 10, 0% of saturation) at a flow rate of 2 ml/min, and each gradient was eluted with two fold column volume of buffer. The purified PoAbf62A was analyzed by sodium dodecyl sulfate–polyacrylamide gel electrophoresis (SDS-PAGE) on 10% separating gel (Laemmli 1970), and protein concentrations were determined by using the method of Bradford with bovine serum albumin (BSA) as the standard (Bradford 1976).

### Biochemical characterization

Specific activity was measured in 200 μl of 20 mM Na-acetate buffer (pH 4.5) containing 10 mM *p*NPαAra*f* and 2 μg recombinant PoAbf62A. After incubating at 37 °C for 30 min, the reaction was stopped by adding 50 μl Na_2_CO_3_ (0.5 M), and the released *p*-nitrophenol was measured spectrophotometrically at 405 nm by using a BioTekELx808 microplate reader (Winooski, VT, USA). The reaction mixture without enzyme was used as a blank. One unit (U) of enzyme activity was defined as the amount of enzyme releasing 1 μmol/min of *p*-nitrophenyl under the assay conditions.

The effect of pH on PoAbf62A activity was determined at different pHs ranging from 2.0 to 11 at 37 °C for 30 min using *p*NPαAra*f* (10 mM) as substrate. The pH stability was investigated under standard assay conditions following incubation of purified PoAbf62A for 24 h at 4 °C in the buffers without substrate. The optimum temperature was determined by measuring enzymatic activity at the optimal pH at temperatures ranging from 20 to 80 °C. Thermostability was assessed by incubating the enzyme at different temperatures (30–45 °C) for up to 100 min. Residual activities were assayed using *p*NPαAra*f* as the substrate at 37 °C for 30 min. The initial activity was defined as 100%.

### Substrate specificity

Substrate specificity was determined using various *p*NP-glycosides as substrates, and the reaction was carried out in 200 μl of 20 mM Na-acetate buffer (pH 4.5) containing 10 mM substrates and 2 μg recombinant PoAbf62A. Hydrolytic activity towards polysaccharides was determined at 37 °C in Na-acetate buffer, pH 4.5, with 0.5% (wt/vol) polysaccharides as substrates and 5 μg PoAbf62A. After incubation for the appropriate reaction time, liberated reducing sugars were measured by the method of Somogyi (Somogyi 1952). To determine hydrolyzates of different arabinose-containing polysaccharides by HPAEC, the reaction mixture containing 50 μl of a 4 mg/ml substrate solution, 140 μl of 20 mM Na-acetate buffer (pH 4.5), and 10 μl of PoAbf62A (100 μg/ml) was performed for 12 h at 37 °C, and the enzymatic productions were analyzed by using high-performance anion-exchange chromatography (HPAEC).

### Mode of action

The mode of action of recombinant PoAbf62A was examined using sugar beet arabinan as substrate and analyzed by methylation and ^13^C NMR. Sugar beet arabinan (20 mg) was treated with PoAbf62A for 12 h in conditions described above. The vacuum-dried sugar beet arabinan and enzymatic product (10 mg) were methylated twice based on the method of Hakomori (Hakomori 1964). Methylated samples were hydrolyzed and reduced with sodium borohydride and acetylated. The partially methylated alditol acetates were analyzed by gas chromatography–mass spectrometry (GC–MS). In order to further assess the mode of action of PoAbf62A, the original sample and enzymatic product (10 mg) were exchanged in D_2_O and analyzed by using ^13^C NMR spectra on a Bruker Avance 600 MHz spectrometer.

### HPAEC analysis

Products were analyzed by HPAEC using a Dionex ICS 5000 system (Dionex Corp, Sunnyvale, CA, USA) with a CarboPac PA-200 analytical column (3 × 250 mm, P/N062896) and pulsed amperometric detection. Samples were eluted at a flow rate of 0.5 ml/min with 0.05 M NaOH for 10 min followed by a linear gradient from 0 to 0.1 M Na-acetate in 0.05 M NaOH for 20 min and finally were eluted by 0.2 M NaOH and 0.5 M Na-acetate for 10 min.

### Methylation analysis

Methylated samples of sugar beet arabinan and its products were analyzed by GC–MS with a Technologies 7890B GC and 5977B MSD equipped with a DB-1 capillary column (0.25 mm × 30 m). The conditions of the GC column were as follows: initial temperature 120 °C for 1 min, then 3 °C/min to 210 °C for 2 min, and then 10 °C/min to 260 °C for 4 min; the injection temperature is 250 °C. Nitrogen was used as a carrier gas and maintained at 1.2 ml/min. The percentage of the methylated sugars was calculated as ratios of the peak areas.

### ^13^C NMR spectra

^13^C NMR spectra were obtained using a Bruker Avance 600 MHz spectrometer (Bruker Inc., Rheinstetten, Germany) operating at 150 MHz for carbon. Samples (10 mg) were dissolved in D_2_O (99.8%, 0.5 ml). Chemical shifts were given in ppm with acetone as the internal chemical shift reference.

### Nucleotide sequences

The sequences for the 18S rDNA and PoAbf62A gene from *P. oxalicum* sp. 68 were submitted to GenBank with accession numbers GU078431 and MG874674, respectively.

## Results

### Cloning, expression and purification of recombinant PoAbf62A

The protein sequence of PoAbf62A from *P. oxalicum* (gene bank accession code EPS32936.1) predicted by Liu et al. (2013), contains 331 amino acids residues with a theoretical molecules mass of 35.9 kDa. Residues 35–329 have sequence similarity to GH family 62 (CAZy database: http://www.cazy.org/CAZY), and the sequences from 1 to 30 is predicted to be a signal sequence. Based on the nucleic acid sequence, the gene for removing the N terminal signal peptide was cloned into pET-32a (+) (*Nde*I/*Eco*RI). The *E. coli* BL 21 (DE3) was chosen as host cell to produce recombinant PoAbf62A. Unfortunately, PoAbf62A formed inclusion bodies in solution because of its *N*-glycosylation sites. The recombinant plasmid (*poabf62a*-pPIC9K) was therefore transformed into GS115 cells by electroporation, and recombinant PoAbf62A was expressed under control of the alcohol oxidase promotor in GS115 and purified from the culture medium by ammonium sulfate precipitation and column chromatography. Using *p*NPαAra*f* as substrate, enzyme activity was determined by measuring the increase in reaction mixture at 405 nm, and the results of purification are summarized in Table 1. Purified PoAbf62A was obtained with efficiently as detected by SDS-PAGE and use of artificial substrates.

**Table 1:** Summary of purification of recombinant PoAbf62A

Purification step	Volume (ml)	Total protein (mg)^a^	Activity (U)^b^	Specific activity (U/mg)	Purification (fold)	Yield (%)
(NH_4_)_2_SO_4_	20	16.7	3.8	0.56	1.0	100
Phenyl Sepharose 6 Fast Flow (high sub)	2	2.2	1.75	0.80	1.43	46

^a^Protein was quantified according to the Bradford method using bovine serum albumin (BSA) as standard

^b^The activity was reported as activity on *p*NPαAra*f*

### Biochemical characterization of recombinant PoAbf62A

**Fig. 1:**
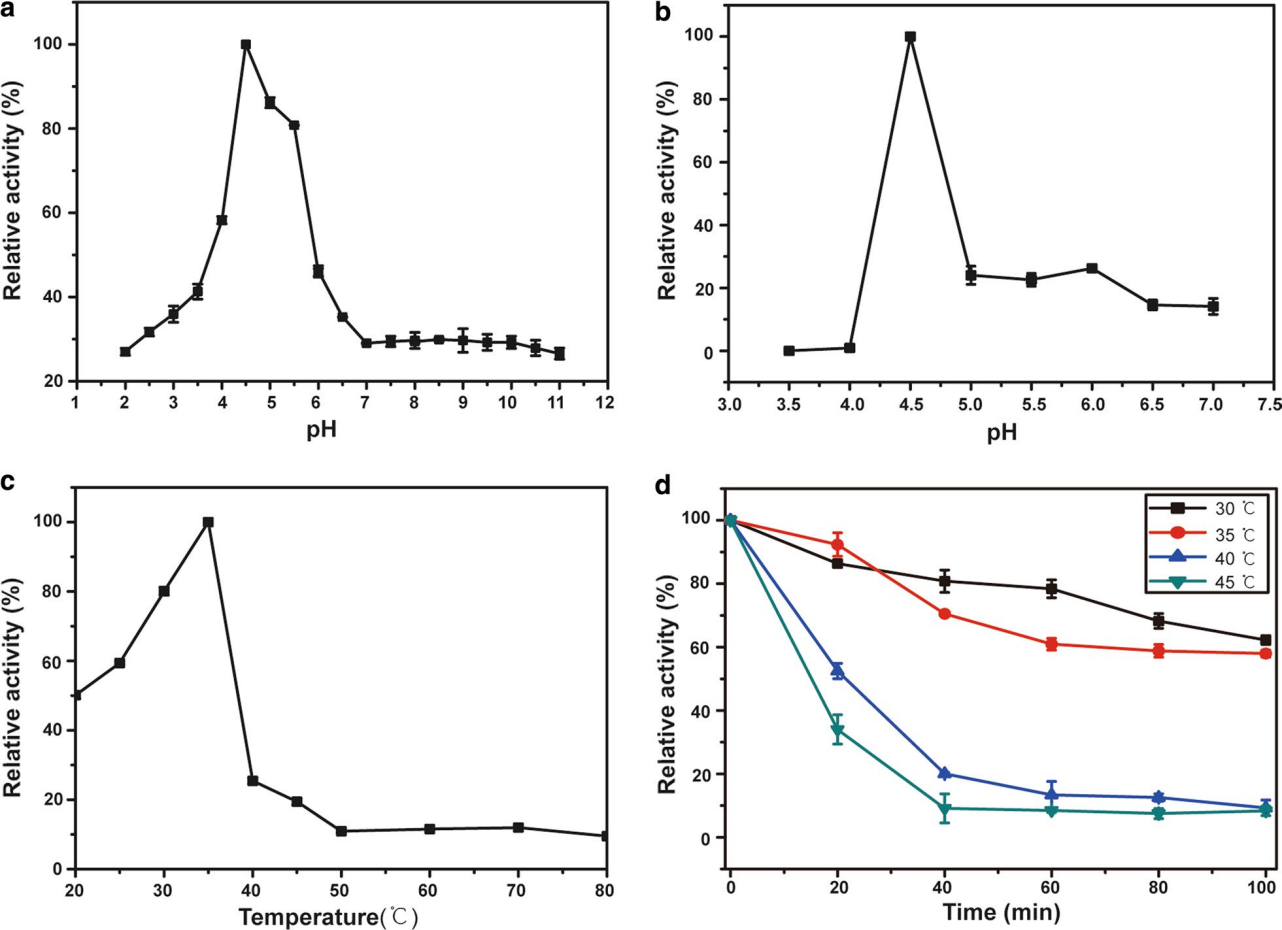
pH and temperature profiles of the purified recombinant enzymes (PoAbf62A) The effect of pH on activity (**a**) and stability (**b**) of PoAbf62A used *p*NPαAra*f* as substrate; and the effect of temperature on activity (**c**) and stability (**d**) of PoAbf62A also used *p*NPαAra*f* as substrate. The activity of the enzyme before incubation was defined as 100%. Results are presented as means ± standard deviations (n = 3)

The hydrolytic activity of recombinant PoAbf62A was examined over a wide pH range from 2.0 to 11.0 using *p*NPαAra*f* as substrate. At pH 4.5, PoAbf62A exhibited its highest activity (Fig. 1a), as well as being quiet stable (Fig. 1b). At this pH, the highest activity was observed at 35 °C (Fig. 1c), whereas proteins stability was reduced at higher temperatures (Fig. 1d). Therefore, for efficiency in biotransformation and structural analysis, we chose the reaction conditions as pH 4.5 and temperature of 35 °C.

### Substrate specificity of recombinant PoAbf62A on *p*NP glycosides and carbohydrates

Activity of PoAbf62A was tested by using different aryl-glycosides. Seven *p*NP glycosides were chosen as substrates to analyze the activity of PoAbf62A. PoAbf62A exhibited high activity towards *p*NPαAra*f*, whereas the enzyme showed little activity with *p*NPβGlc and *p*NPβXyl. PoAbf62A failed to hydrolyze *p*NPαAra*p*, *p*NPαGal, *p*NPβGal and *p*NPαGlc. Therefore, these results demonstrate that PoAbf62A from GH62 family is an α-l-arabinofuranosidase. Using *p*NPαAra*f* as a substrate, the kinetic parameters of purified recombinant PoAbf62A were determined. The *K*_m_, *V*_max_, and *k*_cat_ values were 1.36 ± 0.12 mM, 0.49 ± 0.01 μmol/min/mg, and 0.27 ± 0.01 s^−1^, respectively. The catalytic efficiency *k*_cat_/*K*_m_ was determined to be 0.20 ± 0.02 mM^−1^ s^−1^.

Furthermore, the specificity of PoAbf62A was assayed using different carbohydrates primarily those containing arabinose, and detected by the method of Somogyi. The results showed that PoAbf62A could act on arabinose-containing substrates such as sugar beet arabinan and wheat arabinoxylan which the release content of arabinose were 101 and 13 μg/ml after 12 h incubation with recombinant enzyme, whereas there was almost no impact on linear-1,5-α-l-arabinan, arabinogalactan or xylan. The difference with sugar beet arabinan and linear-1,5-α-l-arabinan is that the former contains branched α-1,2/α-1,3-linked Ara*f*, and we assumed that PoAbf62A specifically degrade α-l-arabinofuranosyl side chains in sugar beet arabinan. In addition, PoAbf62A was effective on wheat arabinoxylan but no on xylan, meaning that PoAbf62A acts on α-l-arabinofuranosyl of arabinoxylan, and not degrade the xylose backbone. Therefore, we assumed that PoAbf62A specifically acts on α-l-arabinofuranosyl side chain.

### HPAEC analysis of the degradation products by PoAbf62A

**Fig. 2:**
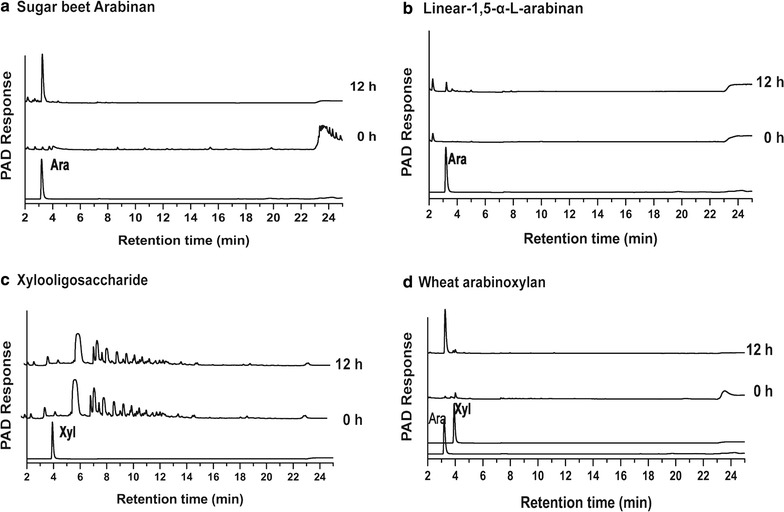
HPAEC-PAD-200 analysis of degradation products of different polysaccharides by PoAbf62A. The reactions were incubated with recombinant enzyme at 37 °C for 12 h, and the experimental conditions are described in the text. The bottom of each graph was the standard sample of arabinose or xylose, and oligosaccharides of the reaction were detected by HPAEC at 0 and 12 h, respectively

So far, our experiments indicated that PoAbf62A is active to artificial substrates and carbohydrates containing α-l-arabinofuranosyl chain. To verify this hypothesis and analyze the products of PoAbf62A from different carbohydrates, we monitored the release of monosaccharide or oligosaccharide using HPAEC. As showed in Fig. 2, PoAbf62A could degrade sugar beet arabinan containing l-arabinofuranosyl side chains and released arabinose (Fig. 2a), whereas it did not work on linear-1,5-α-l-arabinan (Fig. 2b). Therefore, we concluded that PoAbf62A is an exo-α-l-arabinofuranosidase base on the degradetion of sugar beet arabinan that produces only monosaccharide. PoAbf62A also showed activity towards arabinoxylan α-l-arabinofuranose (Fig. 2d), whereas it did not have the function on β-1,4 xylanase (Fig. 2c), because the production of arabinoxylan only had arabinose, and no xylose. Overall, PoAbf62A is an exo-α-l-arabinofuranosidase that degrades branched arabinan from sugar beets to produce arabinose; Moreover, it could also work on l-arabinofuranosyl side chains of arabinoxylan whose arabinoses are linked to C-2 or C-3 of arabinoxylan.

### Mode of action of PoAbf62A

The side chains of sugar beet arabinan are decorated with single α-1,2 or α-1,3-l-arabinofuranose, and some backbone residues are doubly substituted with both α-1,2 and α-1,3 side chains. To further explore the nature of glycosidic bond which PoAbf62A acts, we subjected sugar beet arabinan to methylation and ^13^C NMR spectroscopy analysis post incubation with PoAbf62A. Methylation analysis indicated that the Ara residues mainly existed as terminal, 1,5-, 1,3,5-, 1,2,5- and 1,2,3,5-linked units, in addition containing a small amount of terminal, 1,6-linked Gal residues (Table 2). Among these residues, 1,5-linked Ara*f* was approximately 39.7%, with 28.5% being found at terminal positions. 24.0% of Ara*f* residues were branched at the O-3 position of backbone, and a few of the Ara*f* residues were found as 1,2,5- and 1,2,3,5,-linked units. It is very suitable as a substrate for the study of ABF. As shown in Table 2, the methylated products obviously changed following exposure to PoAbf62A. Five primary methylated products of Ara*f* were detected, including 1,5- (58.9%), 1,3,5- (1.3%), 1,2,5- (0.8%) 1,2,3,5- (3.4%) and terminal (7.8%) linked Ara*f* residues. It could be clearly seen that the content of terminal and 1,3,5-linked Ara*f* decreased, while the content of 1,5-linked Ara*f* increase, with the level of reduction being almost the same as the increase. The content of 1,2,5- and 1,2,3,5-linked Ara*f* was not significantly modified. Taken together, this suggests that PoAbf62A can specifically hydrolyze α-1,3-linked Ara*f* chains present in sugar beet arabinan.

**Table 2:** GC–MS analysis of the methylated products of sugar beet arabinan and its enzymatic product

Methylated sugars	Type of linkage	Molar ratio (before)	Molar ratio (after)	Mass fragments (m/z)
1,4,5-Tri-*O*-actyl-2,3-di-*O*-methyl-arabinitol	1,5-Linked Ara*f*	39.7	58.9	87,101,117,129,189
1,3,4-Tri-*O*-actyl-2,5-di-*O*-methyl-arabinitol	1,3,5-Linked Ara*f*	24.0	1.3	85,99,117,127,159,201,261
1,2,4-Tri-*O*-actyl-3,5-di-*O*-methyl-arabinitol	1,2,5-Linked Ara*f*	1.0	0.8	87,101,117,129,189,203
1,2,3,4,5-Penta-*O*-actyl-arabinitol	1,2,3,5-Linked Ara*f*	4.2	3.4	85,115,127,145,187,233,289
1,4-Di-*O*-acetyl-2,3,5-tri-*O*-methyl-arabinitol	Terminal Ara*f*	28.5	7.8	87,101,117,129,161
1,5-Di-*O*-acetyl-1-deuterio-2,3,4,6-tetra-*O*-methyl-d-galactitol	Terminal Gal*p*	1.3	1.2	71,87,101,117,129
1,5,6-Tri-*O*-acetyl-1-deuterio-2,3,4-tri-*O*-methyl-d-galactitol	1,6-Linked Gal*p*	1.5	1.0	87,101,117,129,143,161,189,233

**Fig. 3:**
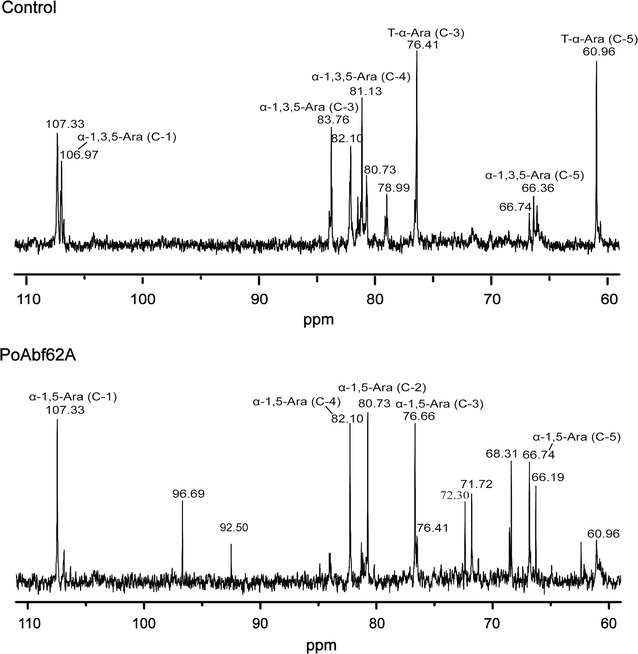
^13^C NMR spectra of the production of sugar beet arabinan and sugar beet arabinan treated with the recombinant PoAbf62A. PoAbf62A (50 mU) was incubated with 2 ml of 0.5% sugar beet arabinan in 20 mM Na-acetate buffer (pH 4.5) at 37 °C for 12 h, and the production was lyophilized and dissolved in 1 ml of D_2_O for analysis

Furthermore, the structure of sugar beet arabinan and its enzymatic product were analysed by ^13^C NMR, as exemplified in Fig. 3. The chemical shifts of major resonances were assigned based on literature values; the anomeric carbon signals of 1,3,5-linked Ara*f* were identified at 106.97 ppm, and the C-3, C-4 of 1,3,5-linked Ara*f* were found to resonate at 83.76 ppm and 81.13 ppm, respectively (Cipriani et al. 2004). The variation in their ratio after hydrolysis indicated that the 1,3,5-linked Ara*f* was cleaved and almost disappeared from there major polymers. Meanwhile, the carbon signal of terminal Ara*f* was also significantly reduced. In contrast, the carbon signals of 1,5-linked Ara*f* were increased as expected. Overall, these results support our proposal that PoAbf62A is an α-1,3-arabinofuranosidase can specifically hydrolyze α-1,3 linked Ara*f* residues in sugar beet arabinan.

## Discussion

Our present study reports on the ability of the recombinant enzyme PoAbf62A of the GH62 family from *P. oxalicum* sp. 68 to hydrolyze arabinose-containing polysaccharides. PoAbf62A exhibits a high specifity for α-1,3-l-arabinan and a much lower activity towards linear-1,5-α-l-arabinan. And the optimal reaction conditions of recombinant PoAbf62A was pH 4.5 and temperature of 35 °C, which showed better adaptability in an acid environment, likely because fungi prefer to grow under mildly acid conditions. This actually makes recombinant PoAbf62A more valuable in application with fruit juices and drinks that clarify under acid condition.

The GH62 family has been characterized with 22 enzymes of the 241 sequences annotated in the CAZy database (Wilkens et al. 2017), GH62 is the one that only contains ABFs in glycoside hydrolase families. The first reported ABFs of GH62 was TrAbf62A which was identified from *Trichoderma reesei* (Poutanen 1988). Like all characterized ABFs from the GH62 family, PoAbf62A can degrade arabinoxylan, and notably, PoAbf62A is also active on l-arabinan, there are only 11 ABFs in the GH62 family that exhibit activity on both arabinoxylan and l-arabinan (Siguier et al. 2014; McCleary et al. 2015), and others were also active on xylan or debranched arabinan. There are only two enzymes more active on sugar beet arabinan than on arabinoxylan (Couturier et al. 2011), and PoAbf62 was more active on sugar beet arabinan in this paper.

The GH62 family is suggested to be involved in plant cell wall penetration (Lanver et al. 2014) and synergistic effects from combination of various hydrolyzing enzymes against hemicelluloses show potential for their use in industrial biotechnology. The wealth of knowledge about the ABFs of the GH62 family helps to develop possible multiple applications. PoAbf62A showed more than 50% identity coverage with five α-l-arabinofuranosidases from *Penicillium* which has been characterized as members of GH62 family. Research has shown that some GH62 ABFs hydrolyze both α-1,2 and/or α-1,3 linked Ara*f* residues in arabinoxylan. ^1^H NMR and hydrolytic fingerprinting revealed that α-l-arabinofuranosidases from *Penicillium chrysogenum* (Sakamoto et al. 2011) and *Penicillium funiculosum* (De La Mare et al. 2013) cleave α-1,2- and α-1,3-bonds that specifically link arabinofuranosyl moieties to single-substituted d-xylosyl residues in arabinoxylan. Meanwhile the enzymes from *Podospora anserina*, *Ustilago maydis* and *Scytalidium thermophilum* are able to efficiently remove the α-1,2 and α-1,3-l-arabinosyl substituents from arabinoxylan which is supported by the crystallographic data (Siguier et al. 2014; Kaur et al. 2015). ABFII (*Aspergillus fumigatus*) and CcAbf62A (*Coprinopsis cinerea*) are specific for α-1,2- and/or α-1,3- bonds of arabinoxylan and sugar beet arabinan, while it did not further explain the mode of action by NMR or crystal (Pérez and Eyzaguirre 2016; Hashimoto et al. 2011). Besides, some ABFs from GH43 family could also release Ara*f* in 1,2- or 1,3-linked groups in doubly substituted Xyl*p* (Cartmell et al. 2011; Pouvreau et al. 2011). While none of them yet characterized ABFs from GH62 family have been shown to specifically hydrolyze α-l-1,3 Ara*f* from sugar beet arabinan. In our present study, we characterized PoAbf62A from *P. oxalicum* sp. 68 which is an exclusive α-1,3-arabinofuranosidase determined by results of HPAEC, Methylation and NMR analysis. Our discovery thus enhances the potential of these ABFs for biotechnological applications, as well as the structural analysis of polysaccharides.

In conclusion, we cloned and characterized α-1,3-arabinofuranosidase PoAbf62A from *P. oxalicum* sp. 68, which is the first GH62 family α-1,3-arabinofuranosidase reported from this strain. PoAbf62A can effectively hydrolyze α-1,3-arabinofuranose groups in sugar beet arabinan and arabinoxylan, releasing arabinofuranose. The enzyme is an α-1,3-arabinofuranosidase with specificity towards arabinose-containing polysaccharides. The discovery and characterization of the α-1,3-arabinofuranosidase PoAbf62A will provide a new tool as a biocatalysts to degrade and analyze the structure of plant cell walls. PoAbf62A will also be helpful for the application of *P. oxalicum* sp. 68 in the saccharification of lignocellulosic material.

## Abbreviations

IPTG: isopropy-β-d-thiogalactoside
D_2_O: heavy water
BGMY: buffered glycerol-complex medium
MD: minimal dextrose medium
G418: geneticin
LB: Luria–Bertani
OD: optical density
